# Identification and Characterization of a Novel Non-Structural Protein of Bluetongue Virus

**DOI:** 10.1371/journal.ppat.1002477

**Published:** 2011-12-29

**Authors:** Maxime Ratinier, Marco Caporale, Matthew Golder, Giulia Franzoni, Kathryn Allan, Sandro Filipe Nunes, Alessia Armezzani, Amr Bayoumy, Frazer Rixon, Andrew Shaw, Massimo Palmarini

**Affiliations:** 1 MRC-University of Glasgow Centre for Virus Research, Institute of Infection, Immunity and Inflammation, College of Medical, Veterinary and Life Sciences, University of Glasgow, Glasgow, United Kingdom; 2 Istituto G. Caporale, Teramo, Italy; University of North Carolina at Chapel Hill, United States of America

## Abstract

Bluetongue virus (BTV) is the causative agent of a major disease of livestock (bluetongue). For over two decades, it has been widely accepted that the 10 segments of the dsRNA genome of BTV encode for 7 structural and 3 non-structural proteins. The non-structural proteins (NS1, NS2, NS3/NS3a) play different key roles during the viral replication cycle. In this study we show that BTV expresses a fourth non-structural protein (that we designated NS4) encoded by an open reading frame in segment 9 overlapping the open reading frame encoding VP6. NS4 is 77–79 amino acid residues in length and highly conserved among several BTV serotypes/strains. NS4 was expressed early post-infection and localized in the nucleoli of BTV infected cells. By reverse genetics, we showed that NS4 is dispensable for BTV replication *in vitro*, both in mammalian and insect cells, and does not affect viral virulence in murine models of bluetongue infection. Interestingly, NS4 conferred a replication advantage to BTV-8, but not to BTV-1, in cells in an interferon (IFN)-induced antiviral state. However, the BTV-1 NS4 conferred a replication advantage both to a BTV-8 reassortant containing the entire segment 9 of BTV-1 and to a BTV-8 mutant with the NS4 identical to the homologous BTV-1 protein. Collectively, this study suggests that NS4 plays an important role in virus-host interaction and is one of the mechanisms played, at least by BTV-8, to counteract the antiviral response of the host. In addition, the distinct nucleolar localization of NS4, being expressed by a virus that replicates exclusively in the cytoplasm, offers new avenues to investigate the multiple roles played by the nucleolus in the biology of the cell.

## Author Summary

Bluetongue is a major infectious disease of ruminants caused by bluetongue virus (BTV), an “arbovirus” transmitted from infected to susceptible hosts by biting midges. Historically, bluetongue has been endemic almost exclusively in temperate and tropical areas of the world. However, in the last decade BTV has spread extensively in several geographical areas causing a serious burden to both animal health and the economy. BTV possesses a double-stranded RNA segmented genome. For over two decades, it has been widely accepted that the 10 segments of BTV genome encode for 7 structural and 3 non-structural proteins. In this study we discovered that BTV expresses a previously uncharacterized non-structural protein that we designated NS4. Although BTV replicates exclusively in the cytoplasm, we found NS4 to localize in the nucleoli of the infected cells. Our study shows that NS4 is not needed for viral replication both in mammalian and insect cells, and in mice. However, NS4 confers a replication advantage to BTV in cells in an antiviral state induced by interferon. In conclusion, we have elucidated a possible route by which BTV can counteract the defences of the host.

## Introduction

Bluetongue is a major infectious disease of ruminants caused by an arbovirus (Bluetongue virus, BTV) transmitted by biting midges (*Culicoides spp.*) [1]–[3]. Historically, bluetongue has been endemic almost exclusively in temperate and tropical areas of the world where the climatic conditions favour both the spread of the susceptible insect vector population and the virus replication cycle within the vector [4]. However, in the last decade BTV has spread extensively in several geographical areas including Southern Europe and also, unexpectedly, in Northern Europe causing a serious burden to both animal health and the economy [5], [6].

From a molecular and structural virology perspective BTV is one of the best understood animal viruses. BTV is a member of the *Orbivirus* genus, within the *Reoviridae* family, and possesses a double-stranded RNA genome formed by 10 segments (Seg-1 to Seg-10) of approximately 19200 base pairs in total [1], [3]. Until now, the BTV genome has been shown to encode for 7 structural and 3 non-structural proteins. The BTV genome is packaged within a triple layered icosahedral protein capsid of approximately 90 nm in diameter [1], [7]–[10]. The outer capsid of the virion is composed by 60 trimers of VP2 and 120 trimers of VP5 [11] and differences within this outer capsid define the 26 BTV serotypes which have been described so far [12], [13]. The outer capsid proteins, and VP2 in particular, stimulate virus neutralizing antibodies which in general protect only against the homologous serotype [14]. The internal core is formed by two layers, constituted by VP3 (sub-core) and the immunodominant VP7 (intermediate layer) [7]. Three minor enzymatic proteins, VP1 (RNA dependent RNA polymerase), VP4 (capping enzyme and transmethylase) and VP6 (RNA dependent ATPase and helicase) are contained within the core that is transcriptionally active in infected cells [15]–[21].

The BTV genome encodes also 3 non-structural proteins: NS1, NS2 and NS3/NS3a. NS1 and NS2 are highly expressed viral proteins and their multimers are morphological features of BTV-infected cells. Multimers of the NS1 protein form tubules (approximately 50 nm in diameter and up to 1000 nm in length) that appear to be linked to cellular cytopathogenicity [22], while NS2 is the major component of the viral inclusion bodies. NS2 plays a key role in viral replication and assembly as it has a high affinity for single stranded RNA and possesses phosphohydrolase activity [23]. NS3/NS3a are glycosylated proteins involved in BTV exit. There are two isoforms of NS3: NS3 and NS3a with the latter lacking the N-terminal 13 amino acid residues [24]–[26].

Therefore, the segmented genome of BTV has been thought to be monocistronic (i.e. ten genome segments encoding for 10 proteins) for almost three decades [27], [28]. Segment 9 however, contains the open reading frame (ORF) encoding VP6 but also a smaller coding sequence in the position +1 reading frame that is present in BTV and some related *Orbiviruses* such as African horse sickness virus and others [29]. Bioinformatic analysis predicts that the BTV “ORFX” encodes for a protein of 77–79 amino acid residues. This putative ORFX is subject to functional constraints at the amino acid level and its level of conservation is higher compared to that of the overlapping VP6. In addition, the ORFX putative AUG initiation codon has a strong Kozak context suggesting that this protein might be translated by leaky scanning [29]. Alternative reading frames are expressed in a variety of RNA viruses and they can play fundamental roles in viral replication and virus-host interaction. In this study, we identified a previously unknown non-structural protein and characterized its biological properties.

## Materials and Methods

### Ethics statement

All experimental procedures carried out in this study are included in protocol number 5182/2011 of the Istituto G. Caporale approved by the Italian Ministry of Health (Ministero della Salute) in accordance with Council Directive 86/609/EEC of the European Union and the Italian D.Igs 116/92.

### Cell cultures

BSR cells (a clone of BHK_21_, kindly provided by Karl K. Conzelmann) were grown in Dulbecco's modified Eagle's medium (DMEM) supplemented with 10% fetal bovine serum (FBS). Bovine foetal aorta endothelium (BFAE) cells were obtained from the Health Protection Agency (HPA) cell culture collection (catalogue number 87022601), and were grown in Ham's F12 medium supplemented with 20% FBS. CPT-Tert cells [30] are sheep choroid plexus cells immortalized with the simian virus 40 (SV40) T antigen and human telomerase reverse transcriptase (hTERT) and were kindly provided by David Griffiths. CPT-Tert cells were grown in Iscove's modified Dulbecco's medium (IMDM), supplemented with 10% FBS. Mammalian cell lines were cultured at 35 to 37°C, in a 5% CO_2_ humidified atmosphere. C6/36 cells are mosquitoes cells established from *Aedes albopictus* and were kindly provided by Richard Elliott. C6/36 cells were grown in Leibovitz's L-15 medium supplemented with 10% FBS and 10% tryptose phosphate broth. KC cells [31](established from *Culicoides sonorensis* larvae) were grown in Schneider's insect medium and were supplemented with 10% FBS. Insect cells were incubated at 28°C.

### Plasmids and antisera

Initially, the open reading frame expressing ORFX (NS4) was amplified by PCR from BTV-10 (GenBank accession number D00509) and cloned into the pCI Mammalian Expression Vector (Promega) resulting into pCI-NS4. The BTV-8 NS4 was cloned into the peGFP-N1 vector (Clontech), resulting in plasmid pNS4-GFP. pNS4_7–77_-GFP, pNS4_13–77_-GFP and pNS4_19–77_-GFP are mutants derived from pNS4-GFP expressing NS4 truncated of the amino terminal 6, 12 and 18 amino acid residues, respectively. pNS4_7–77_-GFP, pNS4_13–77_-GFP and pNS4_19–77_-GFP maintain the methionine and valine residues in position 1 and 2 of NS4. Note that BTV-10 and BTV-1 NS4 are 100% identical at the amino acid level. While BTV-8 and BTV-1 NS4 differ for a single amino acid residue in position 6. The set of BTV-1 and BTV-8 plasmids necessary to rescue these viruses *in vitro* by reverse genetics were obtained following the method recently published by Boyce and colleagues [26]. Briefly, total RNA was extracted from infected cells using Trizol (Invitrogen) according to the manufacturer's instructions. Each BTV genome segment was amplified by RT-PCR using the AccuScript PfuUltra II RT-PCR Kit (Agilent) from either BTV-1 or BTV-8 dsRNA preparations and the resulting PCR products were gel-purified (Qiagen) and cloned into either pUC57 (Fermentas) or pCI. Each BTV segment was cloned downstream of a T7 promoter and upstream of a *BsaI* or *SapI* restriction site. All of the mutants described in this study were obtained using the QuikChange II Site-Directed Mutagenesis Kit (Stratagene), according to the manufacturer's instructions. All plasmids used in this study were completely sequenced before use. Sequences of PCR primers used in this study are available upon request. Antisera used in this study included polyclonal rabbit antisera raised against BTV VP7, NS1, NS2, NS3 and ORFX (NS4) expressed in bacteria as Glutathione S-transferase (GST)-tagged recombinant proteins (Proteintech Group, Inc.). Antiserum against BTV-1 NS4 was raised against a recombinant GST fusion protein including the entire NS4 protein expressed in bacteria. Polyclonal rabbit antiserum against BTV VP6 was kindly provided by Polly Roy as previously described [32]. Antibodies against B23 and γ-tubulin were obtained commercially (Sigma Aldrich).

### Viruses

BTV-8 (IAH reference collection number NET2006/04) was originally isolated from a naturally infected sheep during the 2006 outbreak in Northern Europe [33]. The virus was passaged once in KC cells and once in BHK_21_ cells. The reference strain of BTV-1 was originally isolated at the ARC – Onderstepoort Veterinary Institute (IAH reference collection number RSArrrr/01) and was adapted to cell culture by passaging it twice in embryonated eggs and 9 times in BHK_21_ cells. Both viruses were kindly provided by Peter Mertens. Virus stocks were prepared by infecting BSR cells at a multiplicity of infection (MOI) of 0.01 and collecting the supernatant when obvious cytpopathic effect (CPE) was observed. The supernatants were clarified by centrifugation at 500 *g* for 5 min and the resulting virus suspensions aliquoted and stored at 4°C for short term usage and at −70°C for long term storage. Virus titres were determined by standard plaque assays using BSR or CPT-Tert cells [34].

### Sequence analysis

65 full-length segment 9 sequences representing 24 BTV serotypes were obtained from GenBank. Amino acid conservation plots and secondary structure predictions were obtained using the CLC Genomics Workbench (CLC, Aarhus, Denmark) software and bioinformatics tools available online (the PSIPRED server [http://bioinf.cs.ucl.ac.uk/psipred], and the Network Protein Sequence Analysis (nps@) server, [http://npsa-pbil.ibcp.fr/]).

### Reverse genetics

Recombinant BTVs were rescued by reverse genetics as previously described [26]. Briefly, plasmids containing the genomic segments of BTV-1 or BTV-8 or resulting mutants were linearized with the appropriate restriction enzymes and then purified by phenol-chloroform extraction. Digested plasmids were used as a template for *in vitro* transcription using the mMESSAGE mMACHINE T7 Ultra Kit (Ambion), according to the manufacturer's instructions. ssRNAs were purified sequentially by phenol/chloroform extraction and through Illustra Microspin G25 columns (GE Healthcare Life Sciences), following the manufacturer's protocol. Monolayers of 95% confluent BSR cells grown in 12 well plates were transfected twice with BTV RNAs using Lipofectamine 2000 (Invitrogen). Firstly, 0.5×10^11^ (BTV-1) or 1×10^11^ (BTV-8) molecules of each of the BTV segments encoding VP1, VP3, VP4, NS1, VP6 and NS2 were diluted in Opti-MEM I Reduced Serum Medium containing 0.5 U/µL of RNAsin plus (Promega) and then mixed with Lipofectamine 2000 diluted in Opti-MEM I Reduced Serum Medium. After 25 min of incubation at room temperature, the mixture was added to the cells. 16 to 18 h after the first transfection, the cells were transfected as before but with all 10 BTV segments. 3 to 4 h after the second transfection the cells were overlaid with 2 ml of minimal essential media containing 1.5% agarose type VII and 2% FBS, and monitored for development of plaques. Finally, individual BTV rescued clones were picked through the agarose overlay and used to infect fresh BSR cells in order to obtain a virus stock. Where necessary, BTV dsRNA was extracted from infected cells using Trizol (Invitrogen). The ssRNA fraction was precipitated using lithium chloride, and the harvested dsRNA fraction was precipitated using isopropanol in the presence of sodium acetate.

### Virus growth curves

Growth curves of BTV recombinant viruses used in this study were derived in cells infected at a MOI of 0.05 and testing for the presence of infectious virus in supernatants collected at 8, 24, 48, 72 and 96 h post-infection. Virus growth was also assessed in cells in the presence of 1000 antiviral units/ml (AVU/ml) of interferon *Tau* (IFNT) or universal type I interferon (UIFN). Recombinant ovine IFNT was kindly provided by Tom Spencer. IFNT was produced in *Pichia pastoris* and purified as described previously [35]. Universal type I Interferon (UIFN) was obtained from PBL InterferonSource. BFAE cells and CPT-Tert cells were treated with 1000 AVU of IFN 20 h prior infection with BTV recombinants at a MOI of 0.1 (BFAE and CPT-Tert), 0.01 or 0.001 (CPT-Tert). Two hours after infection, the medium was replaced and the cells maintained in the presence of either IFNT or UIFN at the original concentration. Cell supernatants were collected at 24, 48 and 72 h post-infection, centrifuged for 5 min at 500 *g* in order to pellet cell debris and virus infectivity was subsequently titrated by endpoint dilution analysis on BSR cells. Viral titers were calculated by the method of Reed & Muench and expressed as log_10_ TCID_50_/ml [36]. Each experiment was performed two to three times, each time in duplicate, using different stocks for each virus.

### Interferon protection assay

CPT-Tert cells were plated in 24-well plates and treated for 20 h with 1000 AVU/ml of IFNT or UIFN and then infected with either BTV-1 or BTV-8 at different MOIs (0.1, 0.01 and 0.001). The medium was replaced 2 h after infection and the cells maintained in the presence of either IFNT or UIFN at the original concentration. At 72 h post-infection, the cells were washed once with phosphate buffered saline (PBS; pH 7.4) and stained for 16 h using a 0.5% crystal violet/10% formaldehyde solution. We used Image-Pro Plus (MediaCybernetics), in order to quantify in each well the percentage of the monolayer that was disrupted after BTV replication. Results were expressed as the percentage of destroyed monolayer by calculating for each well the following formula: (number of pixels above background: total number of pixels times) X 100.

### Transfections

BSR cells were transfected with 0.6–1.8 µg of either pCI-NS4, pNS4-GFP or derived deletion mutants, using Lipofectamine 2000 (Invitrogen) according to the manufacturer's instructions.

### Western blotting

For western blot analyses of intracellular proteins, cells were lysed by standard techniques as described previously [37]. For viral pellet analysis, cell supernatants were collected and viral particles concentrated 200 times by ultracentrifugation as previously described [38]. Protein expression was assessed by sodium-dodecyl-sulfate polyacrylamide gel electrophoresis (SDS-PAGE) and western blotting using the various antisera as indicated above. Membranes were incubated with a horse radish peroxidase-conjugated secondary antibody (GE Healthcare Life Sciences) and developed by chemiluminescence using Amersham ECL Plus Western Blotting Detection Reagents (GE Healthcare Life Sciences).

### Confocal microscopy

Experiments were performed using BSR, BFAE, CPT-Tert or C6/36 cells cultured in two-well glass chamber slides (Lab-Tek, Nalge Nunc International). Cells were either transfected with appropriate plasmids or infected with various BTV strains at a MOI between 0.01 and 1.5. Cells were washed with PBS and fixed with 5% formaldehyde for 15 minutes. The fixed cells were then processed as described previously [39] and incubated with the appropriate antisera. Secondary antibodies were conjugated with Alexa Fluor 488 (Invitrogen, Molecular Probes) or Alexa Fluor 594 (Invitrogen, Molecular Probes). Slides were mounted using VECTASHIELD Mounting Medium with DAPI (4′,6-diamidino-2-phenylindole, Vector Laboratories). Slides were analysed and images collected using a Leica TCS SP2 confocal microscope.

### Electron microscopy

BSR cells were infected with BTV-1, BTV-8 or the corresponding deletion mutants at a MOI of 0.05 in 35 mm dishes. At 24 h post-infection, cells were fixed using cold 2.5% gluteraldehyde and 1% osmium tetroxide. Cells were subsequently pelleted through 1% SeaPlaque agarose (Flowgen), dehydrated using a graded alcohol series and embedded in Epon 812 resin, followed by cutting and analysis in a Joel 1200 EX II electron microscope.

### *In vivo* pathogenicity studies

Animal experiments were carried out at the “Istituto G. Caporale” (Teramo, Italy) following local and national approved protocols regulating animal experimental use.

**Study 1.** Litters of 3-day old NIH-Swiss mice (n = 8–12), were inoculated intra-cerebrally with 10^3^ TCID_50_ of either BTV-1, BTV-8, BTV-1ΔNS4 or BTV-8ΔNS4. Mock-infected controls included litters inoculated with tissue culture media. Mice were euthanized at three weeks p.i. or earlier if showing advanced clinical signs of encephalitis. For each virus, two litters were inoculated using two different virus preparations.

**Study 2.** Age-matched adult transgenic mice, deficient in the type I interferon (IFN) receptor [IFN Alpha Ro/o IFNAR^(−/−)^ 129/Sv], were inoculated intraperitoneally with 100 PFU of either BTV-1, BTV-8, BTV-1ΔNS4 or BTV-8ΔNS4. For each virus, two groups (n = 5) of mice were inoculated using two different virus preparations. Survival plots were constructed using data collected from two experimental groups (n = 10) with the exception of a mock infected group that was constituted by a single group of 6 mice. Formalin-fixed and paraffin-embedded brains tissue sections from inoculated (and mock inoculated) mice were used in immunohistochemistry. Sections (4–6 µm) were examined for the presence of BTV NS4 using a polyclonal NS4 antiserum and the EnVision (DAKO) detection system.

## Results

### BTV expresses a previously uncharacterized non-structural protein that localizes in the cell nucleolus

**Figure 1:**
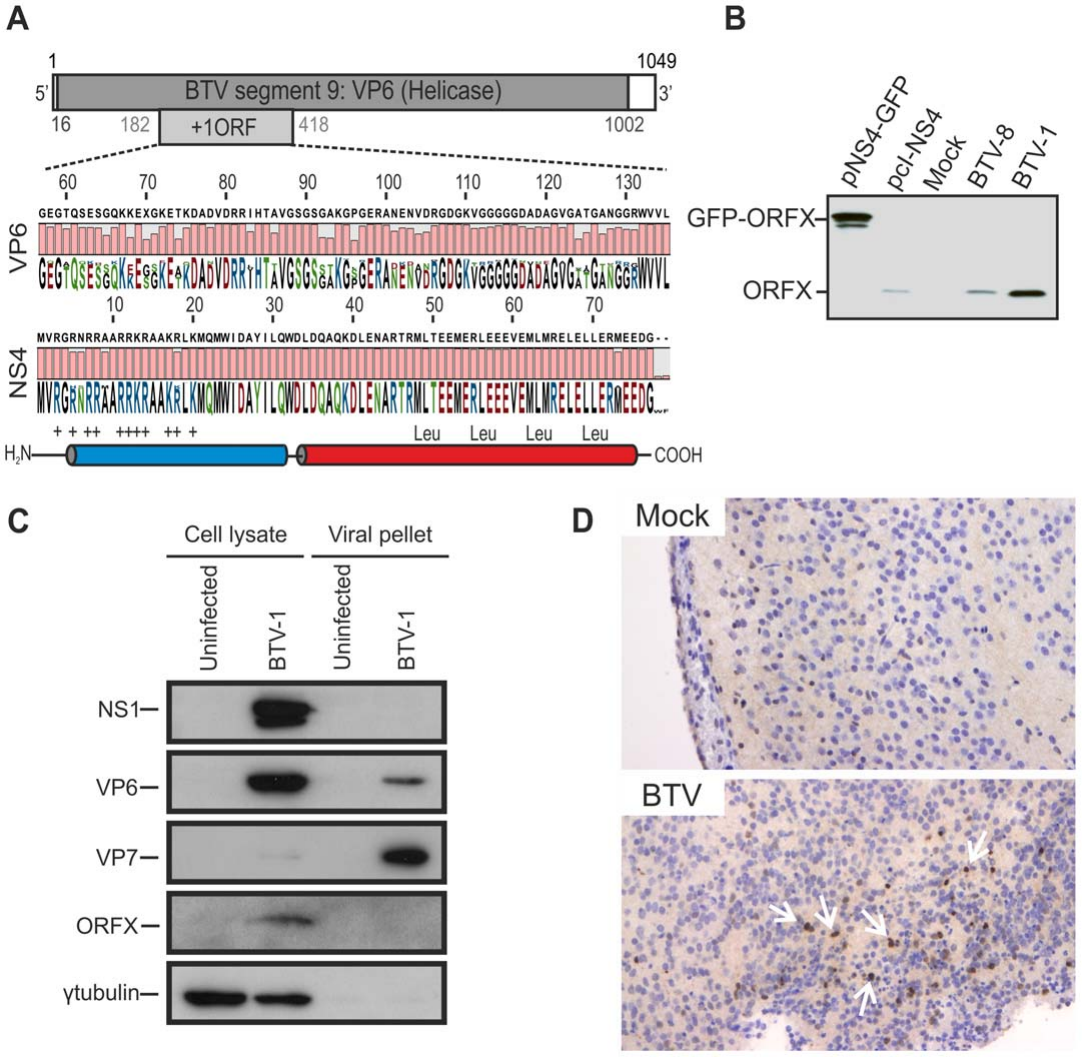
BTV expresses a fourth non structural protein (NS4). (A) BTV segment 9 (1049 base pairs). The VP6 protein (dark gray) is encoded by nucleotides 16 to 1002. The NS4 coding sequence is located in the +1 open reading frame (ORF) between nucleotides 182 to 418. VP6 (residues 57 to 135) and NS4 (residues 1 to 79) amino acid conservation plots are shown. NS4 secondary structure prediction indicated the presence of two putative α-helices, drawn in blue and red. The N-terminal domain (blue) is highly basic and the C-terminal domain (red) contains a conserved leucine zipper motif. (B) Western blotting of cellular extracts (lysate) of BSR cells either transfected with 1.8 µg of plasmid expressing NS4 alone (pcI-NS4) or in fusion with eGFP (peGFP-NS4), or infected by BTV-8 or BTV-1 at a MOI of 0.01. Cells were analyzed 36 h post-transfection or infection and blots were incubated with NS4 antiserum. (C) Western blots of viral pellets and cell protein extracts of BFAE cells infected by BTV-1 at a MOI of 0.05. Samples were analyzed at 48 h post-infection by SDS-PAGE and western blotting employing antisera against NS1, VP6, VP7, ORFX (NS4) and γ-tubulin as indicated. (D) Immunohistochemical detection of NS4. Immunohistochemistry was performed as described in Materials and Methods in brain tissue sections of mice inoculated with BTV-8 72 h post-infection using an antiserum against NS4. Cells expressing NS4 are stained brown as indicated by white arrows. No expression of NS4 is detected in negative control mice mock-inoculated with cell culture media.

The BTV genome is formed by 10 segments. Segment 9 contains an open reading frame (ORF) between nucleotides 182 and 418 (Figure 1A) in position +1 with respect to the major ORF expressing VP6 [29]. *In silico* analysis showed that this extra ORF is highly conserved and encodes a putative protein of 77–79 amino acid residues. A stretch of 11 basic amino acid residues is present in the N terminal portion of the protein (residues 3 to 20). In addition, there are two putative alpha helices. Of note, the C terminal helix (residues 34 to 75) contains a conserved leucine zipper domain, with leucine residues at positions 49, 56, 63 and 70 (Figure 1A).

We generated a polyclonal antiserum towards ORFX, in order to assess whether BTV expressed this previously uncharacterized protein. We detected ORFX in BSR cells infected with either BTV-8 or BTV-1 by western blotting (Figure 1B). Controls included BSR cells transfected with plasmids expressing ORFX either in its native form, or with eGFP fused to its C terminus. These data confirm that BTV expresses a protein encoded by an alternative reading frame located in segment 9.

We subsequently investigated whether ORFX was a structural or non-structural protein. We infected BFAE cells with BTV-1 and analysed supernatants (containing viral particles) and total cellular protein extracts. Unusually for mammalian cell lines, BFAE cells show very little BTV induced cytopathic effect (CPE), thus facilitating the efficient discrimination between all BTV proteins present in the cellular fraction and the structural proteins present in purified and concentrated viral particles released from infected cells. By western blotting, we detected NS1 and ORFX in the cellular fraction, while VP7 was abundantly present in the viral fraction (concentrated by ultracentrifugation) and barely visible in the cellular fraction (Figure 1C). We obtained the same results by infecting C6/36 mosquito cells (data not shown). We detected VP6 in both the cellular and the viral fraction (Figure 1C). Interestingly, unlike VP7, VP6 appeared to be relatively more abundant in cell lysates compared to the viral pellets, suggesting that there is an intracellular pool of this protein that is not incorporated in the BTV virions.

The absence of ORFX in the viral pellet strongly suggested that this is a non-structural protein expressed by BTV. In light of these data, we designated this protein NS4. NS4 was also expressed *in vivo,* as shown by immunohistochemistry of brain sections of mice inoculated intracerebrally with BTV (Figure 1D).

### NS4 localises predominantly in the nucleoli of transfected and infected cells

**Figure 2:**
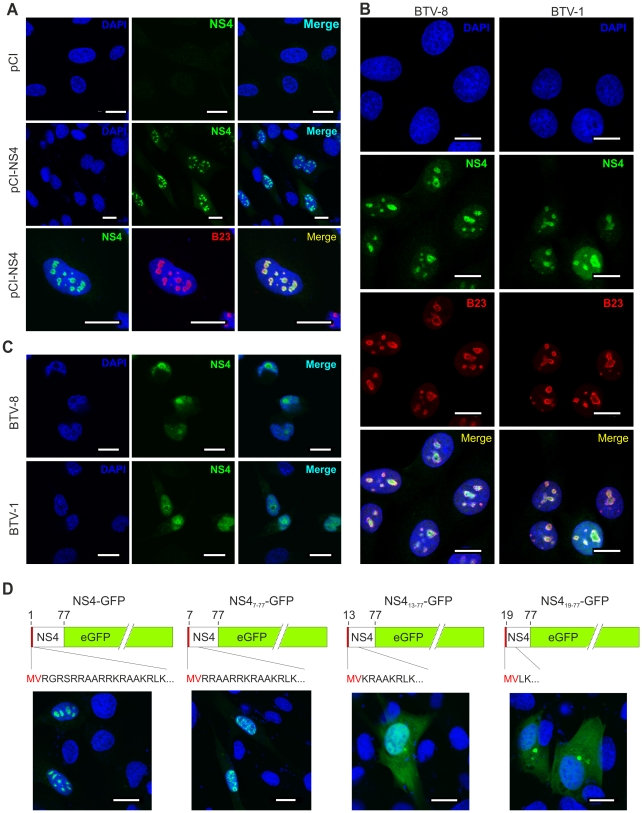
NS4 localises in the nucleoli of transfected and infected cells. (A) Confocal microscopy of CPT-Tert cells transfected with pCI-NS4 or empty pCI as a control. At 24 h post-transfection, cells were fixed and analyzed by immunofluorescence using antibodies against NS4 and as indicated the nucleolar marker B23 with the appropriate conjugated secondary antibodies as described in the Materials and Methods. Scale bars correspond to 18 µm. (B) Confocal microscopy of BSR cells infected by BTV-1 and BTV-8 at a MOI of 0.01. At 24 h post-infection, cells were fixed and analyzed by immunofluorescence as indicated in A. Scale bars correspond to 14 µm. (C) Confocal microscopy of C6/36 cells infected by BTV-1 and BTV-8 at a MOI of 0.05. At 48 h post-infection, cells were fixed and analyzed by immunofluorescence as for expression of NS4 as indicated in panel A. Scale bars correspond to 11 µm. (D) Confocal microscopy of CPT-Tert cells transfected with pNS4-GFP or the truncated mutants indicated above each panel. The red box corresponds to the first two amino terminal amino acid residues of NS4 that were maintained in all mutants. At 24 h post-transfection, cells were fixed and analyzed by immunofluorescence. Scale bars correspond to 18 µm.

By confocal microscopy of cells transiently transfected with pCI-NS4, we observed that NS4 localized mainly in the nucleus (Figure 2A) where it showed a strong co-localization with the nucleolar marker B23 [40]. Importantly, cells infected with either BTV-1 or BTV-8 also showed a strong nuclear co-localisation between NS4 and B23 [40] (Figure 2B). We also observed NS4 to localise in the nucleus of the C6/36 insect cells (Figure 2C).

NS4 does not have a canonical nuclear localization signal (NLS) but possesses a stretch of basic amino acid residues, at the amino terminus portion of the protein, that could drive nuclear localization [41] (Figure 2D). We constructed an NS4 expression plasmid (pNS4-GFP) and a series of deletion mutants (pNS4_7–77_-eGFP, pNS4_13–77_-eGFP and pNS4_19–77_-eGFP) lacking the 6, 12 and 18 amino terminal residues, respectively. pNS4-GFP and pNS4_7–77_-eGFP transfected cells showed a strong nuclear localization of NS4. On the other hand, NS4 showed a predominantly cytoplasmic localization in cells transfected with either pNS4_13–77_-eGFP or pNS4_19–77_-eGFP. These data suggest that the amino terminal basic domain of NS4 may play an important role in the nuclear localization of this protein.

**Figure 3:**
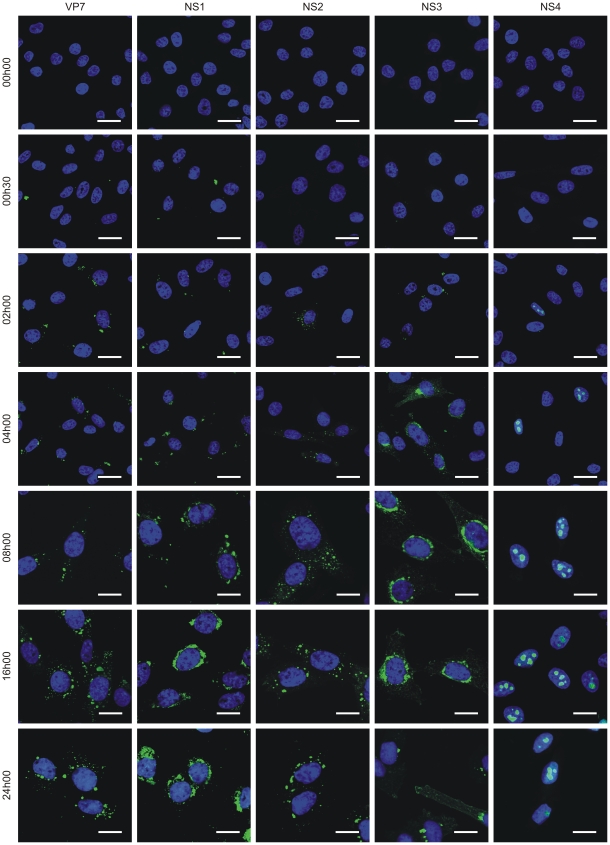
NS4 expression profile. Confocal microscopy of BFAE cells infected with BTV-1 at a MOI of 1.5. Cells were fixed before infection (0 h) and at 0 h30, 2 h, 4 h, 8 h, 16 h and 24 h post-infection and processed for immunofluorescence using antibodies against VP7, NS1, NS2, NS3 and NS4 with an Alexa Fluor 488 secondary antibody as described in the Materials and Methods. Scale bars correspond to 21.16 µm for 0 h to 4 h post-infection panels, and 13.6 µm for 8 h to 24 h post-infection panels.

Interestingly, BFAE cells infected with BTV-1 revealed that NS4 expression was evident as early as 2 hours post infection, similar to that observed for other BTV structural and non-structural proteins (Figure 3).

### NS4 is dispensable for BTV replication

**Figure 4:**
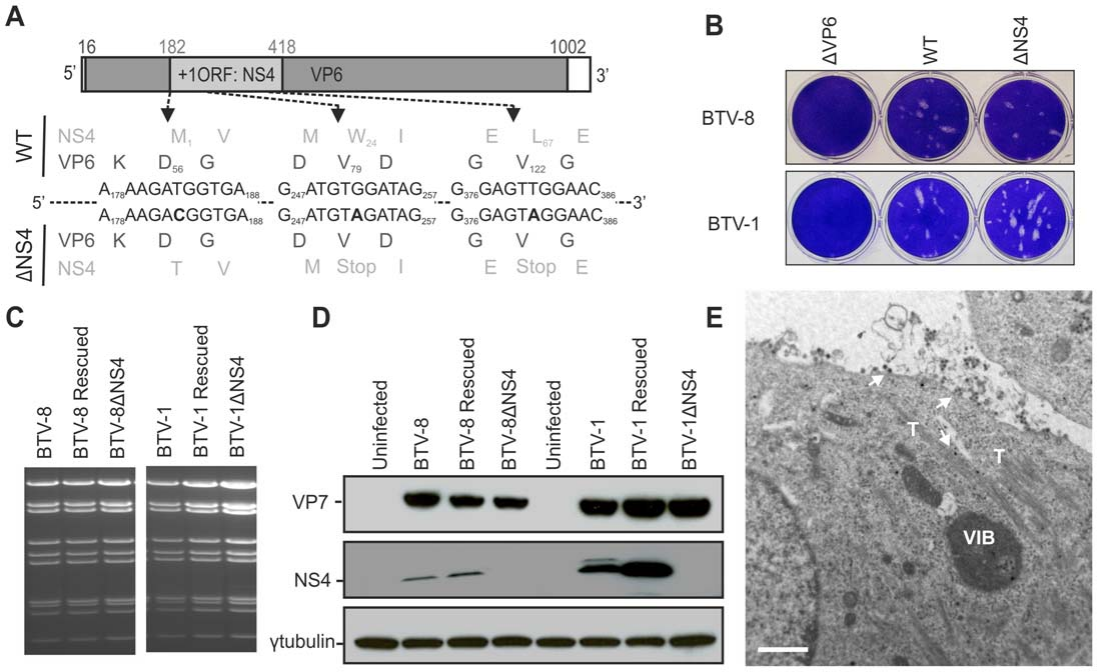
Generation of ΔNS4 Bluetongue viruses by reverse genetics. (A) BTV segment 9 open reading frames. VP6 amino acid residues are written in black, NS4 amino acid residues are written in grey. The nucleotides at positions 183 (T), 252 (G) and 381 (T) were mutated to C, A and A, respectively (bold). Note that whilst these mutations do not change any amino acid residues of VP6, they remove the initiation codon of NS4 (position182) and introduce two stop codons into the NS4 coding sequence at amino acid positions 24 and 67. (B) Transfected BSR cells with BTV transcripts generated *in vitro* (0.5×10^11^ molecules per segment for BTV-1 and 1×10^11^ molecules per segment for BTV-8). Cell monolayers were stained using crystal violet at 72 h post-transfection. As negative controls, ΔVP6 assays correspond to using a segment 9 containing a stop codon at position 79 in the VP6 gene. (C) Agarose gel (1.5%) of purified BTV genomic dsRNA. BSR cells infected at a MOI of 0.01 were collected at 72 h post infection and BTV dsRNA was purified as described in the Materials and Methods. 2 µg of dsRNA was loaded in each lane. (D) Western blotting of cellular extracts (lysate) of BSR cells infected at a MOI of 0.01. Cells were analyzed 36 h post-infection and blots were incubated with antisera against VP7, NS4 and γ-tubulin as indicated. Note that the double NS4 band in the BTV-1 sample is not a feature observed consistently. (E) Electron microscopy of BSR cells infected by BTV1-ΔNS4. Note cells display all the major features of BTV-infected cells including NS1 tubules (T), viral inclusion bodies (VIB) and viral particles (arrows). Scale bar = 1 µm.

The data above clearly show that a previously uncharacterized BTV protein, here referred to as NS4, is a non-structural protein that localises to the nucleolus of infected cells. Next, we generated by reverse genetics BTV NS4 deletion mutants in order to assess the requirement of this protein for viral replication. We generated a set of plasmids necessary for the rescue of BTV-1 and BTV-8 and engineered three mutations in the plasmids containing segment 9 of BTV-1 and BTV-8 such that the NS4 initiation codon was removed along with the introduction of two stop codons in the NS4 coding sequence. All the mutations introduced were designed in order to leave the VP6 amino acid sequence unaltered (Figure 4A). As a negative control for BTV rescue, we designed a VP6 deletion mutant with a premature stop codon incorporated into the VP6 coding sequence (position 79). As shown in Figure 4B, viable BTV1-ΔNS4 and BTV8-ΔNS4 were rescued with similar efficiency to the respective wild-type (wt) viruses, upon transfection of RNA transcribed *in vitro* from the appropriate plasmids representing the genomic segments of wt or mutated BTV-1 and BTV-8. As expected, BTV-1ΔVP6 and BTV8-ΔVP6 could not be rescued.

We did not detect any variation in the migration pattern of dsRNA genomic segments extracted from all the wt or the NS4 deletion mutant viruses (Figure 4C). The RNA profiles of both the wt and ΔNS4 rescued viruses were identical to the corresponding profile of the stock viruses from which the segments were originally cloned. For each virus, segment 9 was completely sequenced in order to confirm the presence of the introduced mutations.

We confirmed, by western blotting and confocal microscopy, that the ΔNS4 mutants do not express NS4 but express levels of VP7 and NS2 comparable to the parental wild type viruses. It was also evident that in BSR cells BTV-8 expresses lower amounts of NS4 relative to BTV-1 (Figure 4D and not shown). However, BTV-1 replicates better than BTV-8 in these cells and differences in the steady-state levels of VP7 between these two viruses were also observed (Figure 4D). In cells infected by BTV1-ΔNS4 or BTV8-ΔNS4, we found by electron microscopy all the ultrastructural features of BTV-infected cells (e.g. viral inclusion bodies, NS1 tubules, viral particles) (Figure 4E).

**Figure 5:**
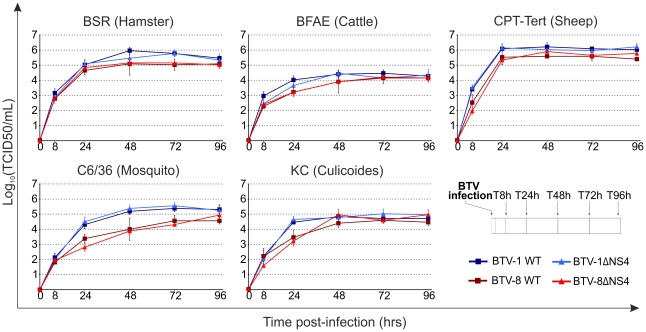
*In vitro* growth properties of rescued WT and ΔNS4 viruses. Growth curves of BTV-8 (dark red, square), BTV8-ΔNS4 (red, triangle), BTV-1 (blue, square) and BTV1-ΔNS4 (light blue, triangle) in cell lines derived from different species. BSR (hamster), BFAE (cattle), CPT-Tert (sheep), C6/36 (mosquito) and KC (*Culicoides*) cells were infected at a MOI of 0.05 and supernatants collected at 8, 24, 48, 72 and 96 h after infection. Supernatants were then titrated on BSR cells by limiting dilution analysis and the virus titers expressed as log_10_ (TCID_50_/ml). In parallel, each virus preparation was also re-titrated by limiting dilution analysis to control that equal amounts of input virus was used in each experiment. Experiments were performed independently twice, each time in duplicate, using two different virus stocks.

We next assessed the replication kinetics of the rescued viruses in a variety of mammalian and insect cell lines, including those corresponding to the natural hosts (sheep and cattle) and vector (midges). All subsequent experiments were performed using the rescued versions of the wt viruses as they represent a more homogenous population and are therefore more directly comparable to the rescued ΔNS4 viruses. Cells were infected with a MOI of 0.05 and supernatants were collected at various times post-infection (Figure 5). No obvious difference was obtained in the replication of wt and ΔNS4 viruses, regardless of the cell lines used in the assay (Figure 5). Interestingly, the cell adapted BTV-1 viruses consistently grew more efficiently *in vitro* than the BTV-8 equivalent, including in cell lines derived from the natural host (BFAE, CPT-Tert and KC).

**Figure 6:**
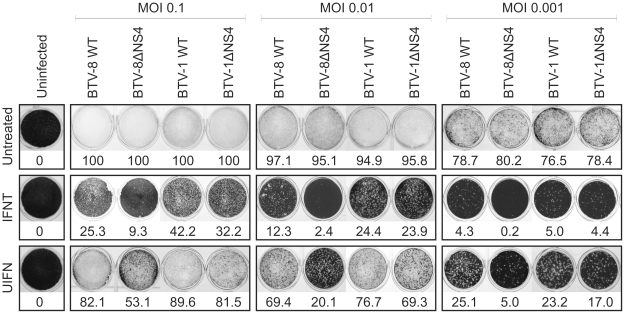
Cytopathic protection assay of CPT-Tert cells monolayer. CPT-Tert cells were treated or mock treated with 1000 AVU/ml of interferon (*Tau,* IFNT or Universal, UIFN) for 20 h prior, and 2 h after, being infected by BTV-8 and BTV-1 (wt and ΔNS4) viruses at different MOIs (0.1, 0.01 and 0.001). Cell monolayers were stained at 72 h post-infection using crystal violet. Values indicated below each well correspond to the relative quantification (in percent) of the disrupted monolayer using Image-Pro Plus (MediaCybernetics, Inc.).

### BTV NS4 confers a replication advantage to BTV-8, but not BTV-1, in mammalian cells treated with interferon

BTV, like most RNA viruses, is a strong inducer of interferon, both *in vivo* in its natural hosts and *in vitro* [42]–[44]. Given that other RNA viruses express proteins that counteract the innate immunity of the host, we hypothesised that NS4 might aid BTV replication in the presence of interferon (IFN). We treated cells with two type I IFNs: IFN *tau* (IFNT) and universal IFN (UIFN). IFNT is secreted by the ruminant conceptus and it is intimately linked to pregnancy recognition signalling and possesses antiviral activity [45] while UIFN is an alpha interferon hybrid constructed from recombinant Human IFNs alpha A and alpha D, and is known to stimulate an antiviral response in a wide variety of mammalian cells.

CPT-Tert cells were pre-treated with IFNT or UIFN for 20 h prior to infection with BTV-1 or BTV-8 (or mock infection) with MOIs ranging from 0.001 to 0.1. Both wt and the ΔNS4 mutants, destroyed 80 to 100% (depending on the MOI used) of the monolayer of infected cells in absence of IFN treatment (Figure 6). On the other hand, pre-treatment with both types of IFN significantly reduced BTV-induced CPE. Interestingly, in the presence of IFN, BTV-8 wt consistently induced a more pronounced CPE than BTV8-ΔNS4. Conversely, only minor differences were observed in the CPE induced by both wt BTV-1 and BTV-1ΔNS4 in the presence of IFN (Figure 6).

Subsequently, we performed multi-step virus growth curves in order to further assess the replication of BTV wt and ΔNS4 in the presence or absence of IFN. CPT-Tert cells were treated with interferon, as described above, and infected at a MOI of 0.01 with wt and mutant viruses. At 24, 48 and 72 h post infection the cell supernatants were collected and the virus titrated in susceptible cells.

**Figure 7:**
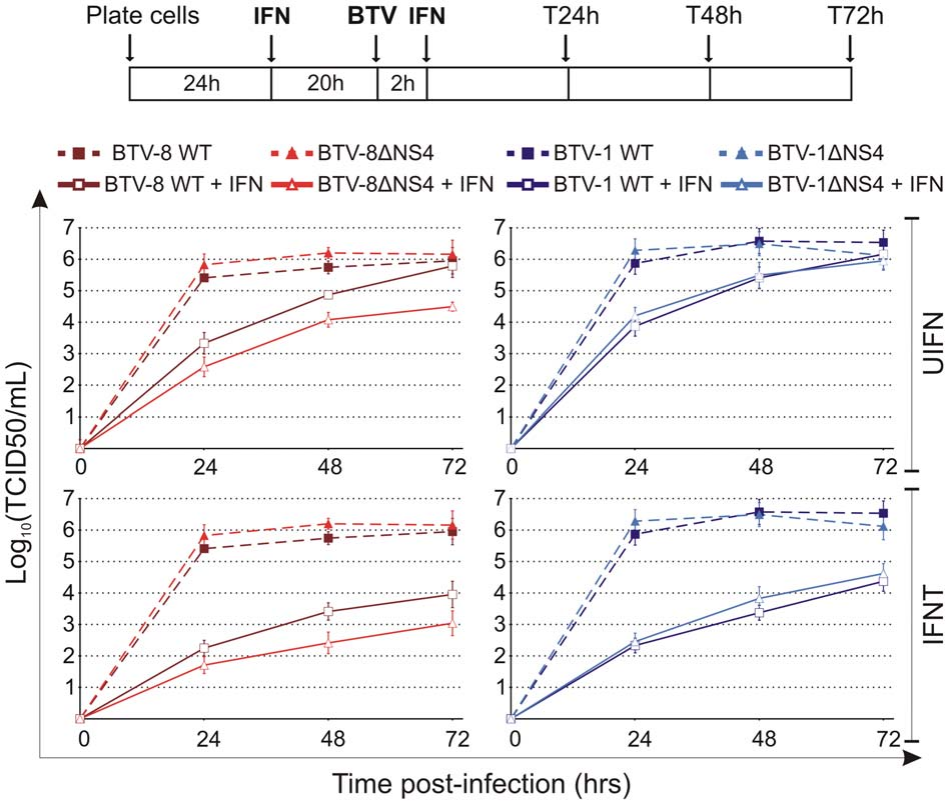
*In vitro* growth properties of rescued WT and ΔNS4 viruses during interferon treatment. CPT-Tert cells were treated (solid line) or mock treated (dashed line) with 1000 AVU/ml of interferon (*Tau,* IFNT or Universal, UIFN) for 20 h prior and 2 h after being infected by BTV-8 (dark red, square), BTV8-ΔNS4 (red, triangle), BTV-1 (blue, square) and BTV1-ΔNS4 (light blue, triangle) viruses. Cells were infected at a MOI of 0.01. Supernatants were collected at 24, 48 and 72 h after infection, and then titrated on BSR cells by limiting dilution analysis and virus titers expressed as log_10_ (TCID_50_/ml). In parallel, each virus preparation was also re-titrated by limiting dilution analysis to control that equal amounts of input virus was used in each experiment. This experiment was performed three times, each time in duplicate.

BTV-8ΔNS4 consistently reached lower titres (approximately 10 to 25 fold) than wt BTV-8 in cells treated with 1000 AVU/ml of either IFNT or UIFN (Figure 7). Similar to what was observed in the IFN protection assays, there was no discernable difference in the replication growth of BTV-1 and BTV-1ΔNS4 after treatment with either IFNT or UIFN. Similar patterns with both BTV-1 and BTV-8 wt and the ΔNS4 mutant viruses where observed when the input viruses were used at a MOI of 0.1 and 0.001 in CPT-Tert (data not shown), or in BFAE cells treated with UIFN and infected at a MOI of 0.1 (data not shown).

**Figure 8:**
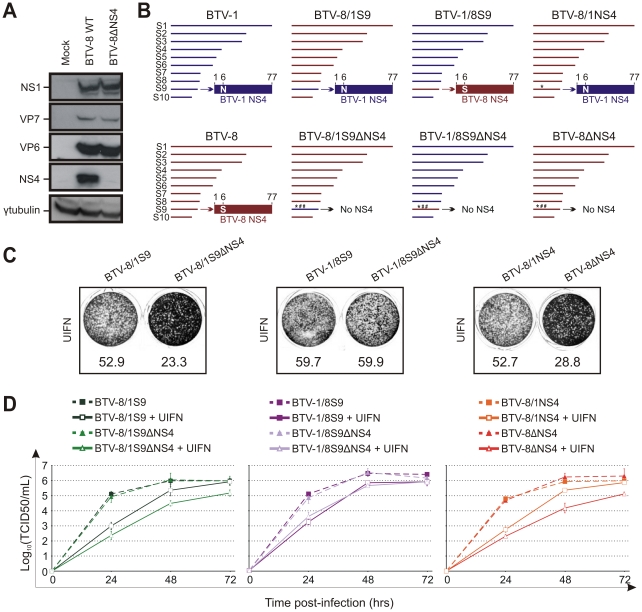
The NS4 of BTV-1 displays similar biological properties to the homologous BTV-8 protein. (A) Western blotting of cellular extracts (lysate) of CPT-Tert cells infected with BTV-8 wt or BTV-8ΔNS4 at a MOI of 0.01. Cells were analyzed 24 h post-infection and blots were incubated with antisera against NS1, VP7, VP6, NS4 and γ-tubulin as indicated. (B) Schematic diagram of the BTV-8/BTV-1 reassortants and mutants used in this study. Note that BTV1 and BTV8 segments/proteins are coloured in blue and red, respectively. * indicates a point mutation, while # indicates the introduction of a stop codon in the NS4 ORF. (C) CPT-Tert cells were treated with 1000 AVU/ml of Universal IFN for 20 h prior, and 2 h after, being infected by the recombinant viruses indicated in the panel using a MOI of 0.01. Cell monolayers were stained 72 h post-infection using crystal violet. Values indicated below each well correspond to the relative quantification of the disrupted monolayer using Image-Pro Plus (MediaCybernetics, Inc.). (D) CPT-Tert cells were treated (solid line) or mock treated (dashed line) with 100 AVU/ml of Universal interferon (UIFN) for 20 h prior and 2 h after being infected by the viruses indicated in the panel. Cells were infected at a MOI of 0.01. Supernatants were collected at 24, 48 and 72 h after infection, and then titrated on BSR cells by limiting dilution analysis and virus titers expressed as log_10_ (TCID_50_/ml). In parallel, each virus preparation was also re-titrated by limiting dilution analysis to control that equal amounts of input virus was used in each experiment. This experiment was performed two times, each time in duplicate.

We next ruled out that the mutations inserted in segment 9 of BTV-8ΔNS4 had a negative effect on VP6 expression (the other protein expressed by segment 9). As shown in Figure 8A, BTV-8 wt and BTV-8ΔNS4 express similar amounts of VP6, reinforcing the notion that the biological differences observed between these two viruses were indeed due to the expression of NS4.

Therefore, the data presented so far suggested that either the BTV-1 NS4 was somewhat defective or that the influence of this protein on viral replication in the presence of IFN varies from strain to strain. In order to discern between these two possibilities, we derived BTV-8 reassortants containing either segment 9 of BTV-1 (BTV-8/1S9) or the ΔNS4 version (BTV-8/1S9ΔNS4). Similarly, we derived BTV-1 reassortants containing the wild type or mutated segment 9 of BTV-8 (BTV-1/8S9 and BTV-1/8S9ΔNS4). In addition, we obtained a BTV-8 recombinant (BTV-8/1NS4) with a single amino acid residue mutated in the NS4 (S to N in position 6) in order to render this protein identical to the homologous BTV-1 protein (Figure 8B). Both cytopathic protection assays and multistep growth assays clearly showed that BTV-8/1S9 replicated more efficiently than BTV-8/1S9ΔNS4 in the presence of IFN (Figure 8C, D). Similar results were obtained with BTV-8/1NS4, which replicated more efficiently than BTV-8ΔNS4 in cells pre-treated with IFN, while no major differences were observed between BTV-1/8S9 and BTV-1/8S9ΔNS4. Collectively, these data strongly indicate that the NS4 of BTV-1 is not defective and can function within the context of BTV-8.

### ΔNS4 BTV mutants are pathogenic in mice models of disease

Next, we assessed the virulence of ΔNS4 BTV mutants in two murine models of bluetongue infection [46], [47]. 129sv IFNAR^(−/−)^ mice, which are deficient in the type I IFN receptor, are susceptible to infection and disease induced by BTV inoculated by various routes [46], [48]. Newborn NIH-Swiss mice inoculated intracerebrally are also susceptible to BTV infection [47]. These models have been previously used to assess BTV virulence [47], [49].

**Figure 9:**
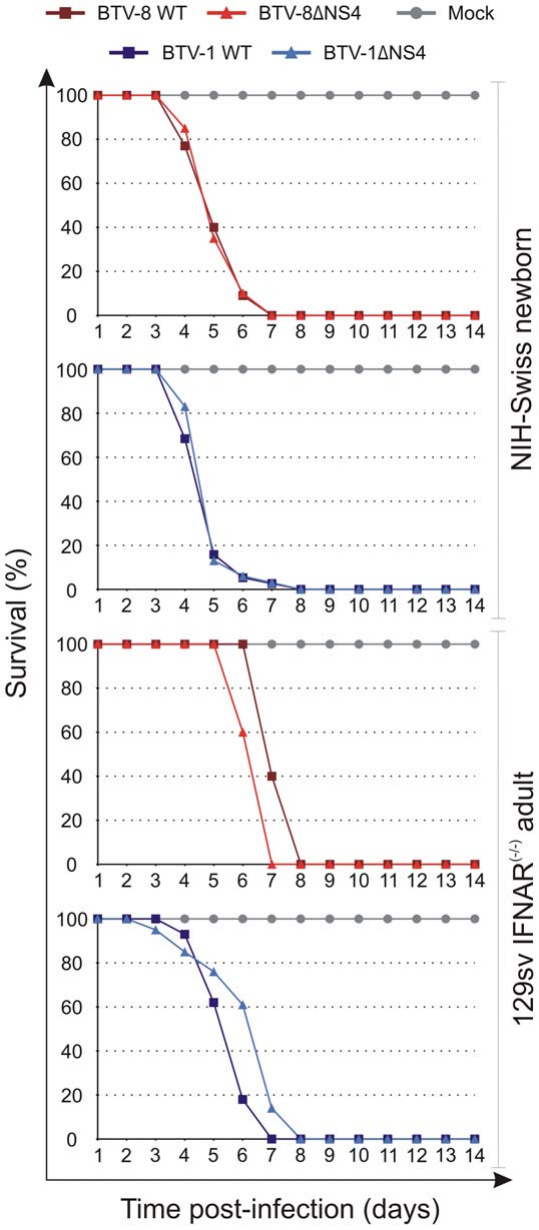
Experimental infection of Swiss new born and IFNAR^(−/−)^ adult mice with wt and ΔNS4 viruses. Survival plots of either 3-days old mice inoculated intracerebrally or adult sv129 IFNAR^(−/−)^ inoculated intraperitoneally with the following viruses: BTV-8 (dark red, square), BTV8-ΔNS4 (red, triangle), BTV-1 (blue, square) and BTV1-ΔNS4 (light blue, triangle) viruses. Mock-infected mice are shown in gray, circle. Mice were killed at two weeks post-inoculation, or earlier, if showing advanced clinical signs of systemic disease.

In this study, we infected 129sv IFNAR^(−/−)^ mice with either BTV-1, BTV-8 or the corresponding ΔNS4 mutants. No major differences were observed in the virulence of wild type and ΔNS4 viruses; all viruses employed in this study killed 100% of the inoculated mice by day 8 post-infection (Figure 9). We also inoculated 3-day old NIH-Swiss mice intracerebrally with the same viruses as above. Once again, both wild type and ΔNS4 viruses were able to kill 100% of the inoculated mice with no major differences in the virulence observed (Figure 9).

## Discussion

In this study we have shown that BTV expresses a previously uncharacterised non- structural protein that favours viral replication in cells in an antiviral state. By constructing deletion mutants by reverse genetics, we showed that NS4 is dispensable for viral replication *in vitro*, both in mammalian and insect cells, and *in vivo* in murine experimental models. However, the coding sequence in the NS4 reading frame of segment 9 is highly conserved in BTV and in related *Orbiviruses* [29], [50], suggesting that it must be essential for the maintenance of BTV in nature. Indeed, we have found that NS4 confers a replication advantage to BTV-8 in cells pre-treated with type I IFN.

We found NS4 to have strong nucleolar localization, although it may shuttle between the nucleolus and cytoplasm and possibly carry out its biological functions in the latter. The nucleolus is a dynamic sub-nuclear structure that plays crucial roles in ribosome subunit biogenesis, the response to cellular stress and cell growth [51], [52]. Several examples of viral proteins targeting the nucleolus have been discovered in recent years [53]. The retroviral Rev and Rev-like proteins for example, shuttle between the nucleolus and cytoplasm, and function as post-transcriptional regulators of viral gene expression [54]–[57]. One of the main functions of these proteins is to facilitate the export of unspliced viral mRNA (transcribed from the proviral DNA copy of the retroviral genome stably integrated in the cell genome) by simultaneously binding an RNA structure in the viral RNA and the karyopherin export factor Crm1 (chromosome region maintenance 1) [58]. Other RNA viruses (including those that replicate exclusively in the cytoplasm) have also been found to possess proteins that target the nucleoli. Examples include, among others, avian infectious bronchitis virus [59], porcine reproductive and respiratory syndrome virus [60], Newcastle disease virus [61], Semliki forest virus [62], dengue virus [63], West Nile virus [64], influenza virus [65], avian reovirus [66] and encephalomyocarditis virus [67], [68] The reasons for the nucleolar targeting of many of these proteins have not always been entirely clear.

The avian reovirus σA protein is a structural protein and is a major component of the inner capsid shell. Although the σA protein localises mainly in viral factories in the cytoplasm of infected cells, it also localizes in the nucleoli [66]. σA has a strong affinity for dsRNA and it may provide protection against the IFN-induced and dsRNA dependent PKR response. Interestingly, σA mutants that do not bind dsRNA are also unable to reach the nucleoli, suggesting that dsRNA binding and nucleolar targeting may be strictly linked [69].

BTV NS4 may also bind nucleic acids but, unlike the reovirus σA, we show strong evidence that NS4 is not a structural protein. Indeed, by western blotting we did not detect NS4 in viral particles but only in lysates of BTV infected cells. In addition, by confocal microscopy we did not detect NS4 in viral inclusion bodies but predominantly in the nucleoli of viral infected cells. We cannot exclude completely that small amounts of NS4, below the limits of detection of our western blotting analysis, are present in viral particles.

The predicted structural features of NS4 resemble those of a transcription factor of the bZip family with a basic domain followed by a leucine zipper motif [70]. Thus, NS4 may function as a nucleic acid binding protein and either repress or enhance transcription of genes linked directly or indirectly to the IFN response of the cell. However, a BTV-8 recombinant virus (BTV-8ΔLZNS4) expressing an NS4 with all the 4 leucine residues forming the putative leucine zipper mutated (into either glutamine or serine) replicated as efficiently as BTV-8 wt in cells pre-treated with IFN (data not shown). Thus, more studies will be necessary to explore this possibility.

The organization of VP6/NS4 ORFs in segment 9 of BTV mirrors that of NSP5/NSP6 in the rotavirus segment 11 [71]. The rotavirus NSP6 is not essential for virus replication but unlike the BTV NS4, does not localize in the nucleus of infected cells [72].

To date, limited information is available on the interplay between BTV and the host innate immune system. BTV has been recognized as a potent inducer of type I IFN in sheep [42], cattle [43] and mice [73]. However, limited data have been available on how BTV induces the IFN response of the cell and, more importantly, what counteracting measures the virus utilises to overcome this response. Our data suggest that BTV may use NS4 to defend itself from the innate immune response of the host given that replication of BTV8-ΔNS4 in cells treated with IFN is 10 to 25 fold less efficient compared to wild type BTV-8. In addition, the cytopathic effect in cells treated with IFN is more pronounced when cells are infected by wild type BTV-8 compared to cells infected by BTV8-ΔNS4.

Viruses have evolved a variety of strategies to evade the host innate immunity [74]. Other dsRNA viruses such as rotaviruses, use different mechanisms (which vary between strains and the type of infected cells) to modulate the type I IFN response. For example, rotaviruses use NSP1 protein to promote the proteasome-dependent degradation of IRF proteins [75]–[77] and mediate repression of NF-kB, resulting in a reduction of IFN induction [78]. Rotaviruses also induce shut off of cellular protein synthesis resulting from the detection of dsRNA by PKR which, in turn is responsible for phosphorylation and consequent inhibition of the eukaryotic translation initiation factor eIF2α [79]. The blocking of host cell protein synthesis is another likely strategy used by some RNA viruses to counteract the IFN response [67], [68]. BTV also blocks host cell protein synthesis early after infection, although the mechanisms underlying this phenomenon are not clear [27].

Interestingly, we found that BTV1-ΔNS4 replicated as efficiently as wild type BTV-1, even in cells treated with IFN. However, the NS4 of BTV-1 appears to possess the same biological properties of the NS4 of BTV-8. Indeed, a BTV-8 reassortant containing the entire segment 9 of BTV-1 (BTV-8/1S9) or a recombinant BTV-8 expressing an NS4 100% identical to the homologous BTV-1 protein (BTV-8/1NS4), maintained the phenotype of wt BTV-8. Thus, it is possible that the role played by NS4 in counteracting the IFN response of the host could vary between different virus strains. It is important to stress that the strain of BTV-8 that we used in this study has been passaged only a few times in culture (once in KC cells and three times in BHK_21_ cells) after isolation from blood of an infected animal. On the other hand, BTV-1 was derived from the “reference” South African strain passaged twice in embryonated eggs and 9 times in BHK_21_. BTV-1 appears to grow slightly faster than BTV-8 in culture, especially at the early time points post infection. Thus, faster replication may help BTV1-ΔNS4 to escape the IFN response of the cell more efficiently, as already suggested for some strains of influenza, and this may render NS4 less critical in these *in vitro* assays [80].

More *in vivo* experiments will be needed in order to determine the role of NS4 in the interplay with the natural host of BTV infection. We observed no differences between wild type BTV-8 and BTV8-ΔNS4 in experimental mouse models, although it remains possible that differences could be identified in sheep. It is possible that NS4 is required for viral replication in insects, although we have established in this study that no differences are observed on the replication of the ΔNS4 mutants in insect cells *in vitro*.

In conclusion, in the present study we have identified a previously uncharacterized non-structural protein of BTV. The identification of this highly conserved protein opens the way to understand finer details of virus-host interaction and pathogenesis. In addition, the distinct nucleolar localization, in a virus that replicates exclusively in the cytoplasm will offer new avenues to understand the various roles played by these organelles in the biology of the cell.
